# On the Lanthanide Effect on Functional Properties of 0.94Na_0.5_Bi_0.5_TiO_3_-0.06BaTiO_3_ Ceramic

**DOI:** 10.3390/ma17081783

**Published:** 2024-04-12

**Authors:** Jacem Zidani, Ilham Hamdi Alaoui, Moneim Zannen, Eriks Birks, Zakaria Chchiyai, Mustapha Majdoub, Bouchaib Manoun, Mimoun El Marssi, Abdelilah Lahmar

**Affiliations:** 1 Laboratory of Physics of Condensed Matter (LPMC), University of Picardie Jules Verne, Scientific, Pole, 33 rue Saint-Leu, 80039 Amiens, France; jacem.zidani@etud.u-picardie.fr (J.Z.); ilham.hamdi.alaoui@u-picardie.fr (I.H.A.); mimoun.elmarssi@u-picardie.fr (M.E.M.); 2 Laboratory of Interfaces and Advanced Materials (LIMA), Faculty of Sciences of Monastir, University of Monastir, Bd. of the Environment, Monastir 5019, Tunisia; moneimchimie2006@yahoo.fr (M.Z.); mustaphamajdoub@gmail.com (M.M.); 3 Institute of Solid-State Physics, University of Latvia, LV-1586 Riga, Latvia; eriks.birks@cfi.lu.lv; 4 Laboratory of Inorganic Materials for Sustainable Energy Technologies (LIMSET), Mohammed VI Polytechnic University (UM6P), Benguerir 43150, Morocco; chchiyai.zakaria@gmail.com; 5 FST, Rayonnement-Matière et Instrumentation, S3M, Hassan First University of Settat, Settat 26000, Morocco; bouchaib.manoun@um6p.ma; 6 Materials Science, Energy, and Nano-Engineering Department, University Mohammed VI Polytechnic, Ben Guerir 43150, Morocco

**Keywords:** sodium bismuth titanate, structural refinement, dielectric properties, ferroelectric properties, piezoelectric properties, photoluminescence

## Abstract

The beneficial effects of lanthanide incorporation into 0.94Na_0.5_Bi_0.5_TiO_3_-0.06BaTiO_3_ (BNT-BT) matrix on its functional properties were investigated. The conventional solid-state method was used for synthesizing samples. The structural refinement revealed that all samples crystallized in R3c rhombohedral symmetry. Raman spectroscopy study was carried out using green laser excitation and revealed that no clear perceptible variation in frequency is observed. Dielectric measurements unveiled that the introduction of rare earth obstructed the depolarization temperature promoted in BNT-BT, the diffusive phase transition decreasing with increasing lanthanide size. Only dysprosium addition showed comparable diffusion constant and dielectric behavior as the unmodified composition. Further, the comparison of the obtained ferroelectric hysteresis and strain-electric field loops revealed that only Dy-phase exhibited interesting properties comparing parent composition. In addition, the incorporation of lanthanides Ln^3+^ into the BNT-BT matrix led to the development of luminescence characteristics in the visible and near infrared regions, depending on the excitation wavelengths. The simultaneous occurrence of photoluminescence and ferroelectric/piezoelectric properties opens up possibilities for BNT-BT-Ln to exhibit multifunctionality in a wide range of applications.

## 1. Introduction

Piezoelectric materials have become increasingly important in various applications such as sensors, actuators, and transducers. However, lead-based piezoelectric materials pose a significant risk to human health and the environment, which has led to the development of lead-free alternatives. Among these alternatives, Na_0.5_Bi_0.5_TiO_3_ (BNT)-based ceramics have gained attention due to their promising piezoelectric properties [1]. BNT is classified as a relaxor ferroelectric with (T_m_) = 320 °C as reported in previous works [2,3]. 

The perovskite structure ABO_3_ is occupied by Bi and Na ions in the A-sites. BNT has an R3c rhombohedral structure at room temperature [4]. Ferroelectric materials, which exhibit dielectric and piezoelectric properties, are widely used in high-performance applications. The emergence of perovskite relaxor ferroelectric materials has opened up fresh avenues across diverse sectors such as medical ultrasonics and energy harvesting [5,6]. In particular, the exceptional piezoelectric properties of perovskite relaxor ferroelectric materials make them suitable for acoustic transducers. Therefore, the development of lead-free piezoelectric ceramics with enhanced electrical and mechanical properties, such as dopant-modified BNT-based ceramics, can lead to the development of new fields of applications [7]. However, the practical application of BNT-based ceramics is limited by their poor electrical and mechanical properties [8]. To overcome these limitations, dopants have been introduced to modify their crystal structure, resulting in enhanced electrical and mechanical properties [9]. 

As already known, it has been previously noted that the morphotropic phase boundary (MPB) holds significant importance in lead-based solid solutions [10]. This is due to the fact that the reported piezoelectric and dielectric properties are at their highest in close proximity to the MPB region, providing ample opportunities for enhancing their practical application. Likewise, the Na_0.5_Bi_0.5_TiO_3_-0.06BaTiO_3_ (BNT-BT) system has gained significant interest due to the presence of the MPB situated between the rhombohedral and tetragonal phases, which occurs near x = 0.06 [11,12]. The addition of BT to NBT at the MPB results in a notable decrease in coercive fields and significant enhancement in piezoelectric properties when compared to pure BNT [8]. 

Recent studies [13,14] have shown that incorporating lanthanide elements into the 0.94NBT-0.06BT system can improve properties that are linked to the specific rare earth element used [15,16,17,18]. The addition of Ln_2_O_3_ to BNT-BT piezoelectric ceramics not only enhances their ferroelectric and piezoelectric properties but is also responsible for fluorescence. This finding suggests a potential application of these ceramics in fluorescent light-emitting devices. However, according to Wu et al. [19], current research lacks a comprehensive and systematic examination of how the doping by different lanthanides impacts the ferroelectric, dielectric, and field-strain properties and the fluorescence luminescence intensity of BNT-BT ceramics. 

These findings emphasize the importance of lanthanides in enhancing the properties of 0.94NBT-0.06BT ceramics. The objective of this study is to examine how the addition of Ln_2_O_3_ influences the structural, dielectric, piezoelectric, optical and ferroelectric characteristics of 0.94BNT-0.06BT ceramics.

## 2. Materials and Methods

The BNT and 0.94Na_0.5_(Bi_1−x_Ln_x_)_0.5_TiO_3_-0.06BaTiO_3_ ceramic doped with lanthanides (x = 1%, Ln = Pr, Nd, Eu, Dy) were prepared by the conventional solid-state technique. We used precursors with high purity such as Na_2_CO_3_ (99%), Bi_2_O_3_ (99%), TiO_2_ (99.9%), BaCO_3_ (99.9%), Pr_2_O_3_ (99%), Nd_2_O_3_ (99%), Dy_2_O_3_ and Eu_2_O_3_ (99%). All these starting reagents are purchased from Alfa Aesar (Karlsruhe, Germany). The powders were weighed and blended based on the stoichiometric ratio, after which they underwent thorough milling in an agate mortar with ethanol to facilitate the grinding. The calcination procedure was carried out at 850 °C for 3 h (heating rate = 300 °C/h). Subsequently, the powders underwent further grinding in an agate mortar. The crushed powders were pressed at 250 MPa into disks of 8 mm in diameter and 1 mm in thickness. Finally, the compressed discs were subjected to sintering at 1100 °C for 3 h, employing the same heating rate.

The structure BNT-BT-Ln was studied at room temperature using a Bruker D8 Advanced diffractometer (CuKα = 1.5406 Å, Karlsruhe, Germany) between 10° and < 2θ and < 80°. The data underwent refinement using the Rietveld refinement method with the FullProf software (version 7.95). The dielectric measurements (εT, tanδ and Cp) were performed at different frequencies using an Solartron Impedance/GAIN-PHASE analyzer SI-1260 (AMETEK scientific instruments, Oak Ridge, TN, USA). in the temperature range of 300–800 K. The microstructure of the ceramics was studied by a scanning electron microscopy (SEM) device (Environmental Quanta 200 FEG, FEI company, Hillsboro, OR, USA). Raman spectra were obtained within the 100–1000 cm^−1^ range utilizing a micro-Raman Renishaw spectrometer LABRAM HRT 4600 HR 800 (Wotton-under-Edge Gloucestershire, UK) with 532 nm laser excitation. Polarization hysteresis loops were assessed using the Sawyer–Tower technique. The dielectric permittivity measurements were conducted on samples that had been previously poled employing an impedance analyzer (TF Analyzer 2000, aixACCT, Aachen, Germany). Poling was carried out at room temperature with a 1 Hz frequency, subjecting the material to a maximum electric field of 75 kV/cm. Luminescence analysis was conducted using photoluminescence spectroscopy (LabRAM HR Evolution), employing various laser wavelengths for excitation across all synthesized samples. Specifically, a laser wavelength of 360 nm was utilized for BNT-BT-Pr^3+^, BNT-BT-Eu^3+^, and BNT-BT-Dy^3+^, while a wavelength of 781 nm was used for BNT-BT-Nd^3+^. 

## 3. Results and Discussion

### 3.1. X-ray Diffraction and Structural Analysis

**Figure 1:**
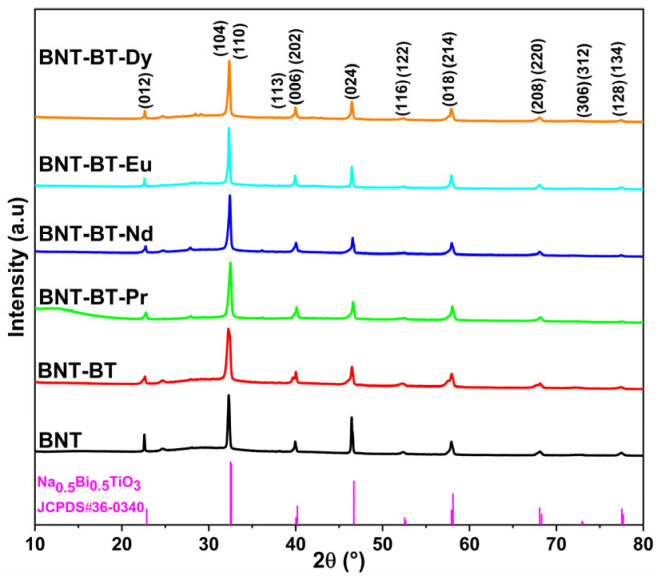
X-ray diffraction profiles of the synthesized BNT, BNT-BT, and BNT-BT-Ln (Ln = Pr^3+^, Nd^3+^, Eu^3+^, Dy^3+^) perovskite ceramics.

The phase purity and structural properties of the elaborated perovskite ceramics are examined by the powder X-ray diffraction (XRD) and Rietveld method. Figure 1 exhibits the room temperature powder XRD profiles of the synthesized Na_0.5_Bi_0.5_TiO_3_ (BNT), 0.94Na_0.5_Bi_0.5_TiO_3_-0.06BaTiO_3_ (BNT-BT), and Ln-doped 0.94Na_0.5_Bi_0.5_TiO_3_-0.06BaTiO_3_ (BNT-BT-Ln, Ln = Pr^3+^, Nd^3+^, Eu^3+^, Dy^3+^) perovskite ceramics. From this figure, all the synthesized compounds exhibit similar XRD patterns indicating a similar structure for all the elaborated compounds with good crystallization. As shown in Figure 1, most XRD peaks of different compounds are indexed to the Na_0.5_Bi_0.5_TiO_3_ (BNT) perovskite structure according to JCPDS card number 36-0340, which crystallizes in the rhombohedral R3c system with the appearance of small impurities at 24.66°, 27.93°, and 36.16°. Thus, all the synthesized BNT, BNT-BT, and BNT-BT-Ln compounds exhibit the polycrystalline ABO_3_-type perovskite structure with R3c rhombohedral distortion.

**Figure 2:**
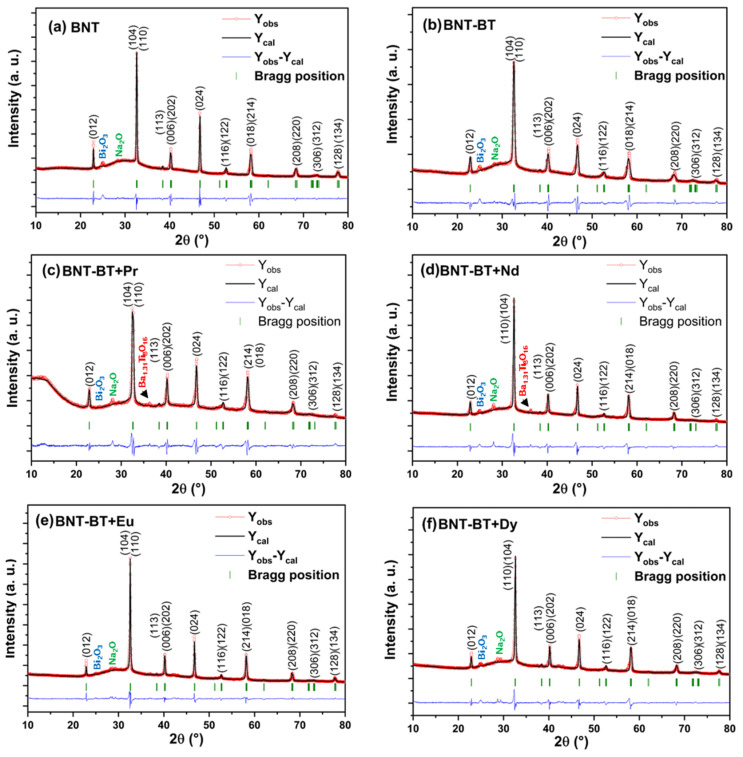
Rietveld refinements of the room temperature powder XRD profiles for the synthesized (**a**) Na_0.5_Bi_0.5_TiO_3_ (BNT), (**b**) 0.94Na_0.5_Bi_0.5_TiO_3_-0.06BaTiO_3_ (BNT-BT), and (**c**–**f**) lanthanides-doped (BNT-BT-Ln, Ln = Pr^3+^, Nd^3+^, Eu^3+^, Dy^3+^) perovskite ceramics using the rhombohedral structure with the space group R3c. The red circles and black lines are the experimental and calculated XRD profiles, respectively. The blue lines and green bars represent the difference (Y_obs_-Ycal) and the Bragg position of (hkl) planes, respectively.

For further investigation and to determine the structural parameters of all the synthesized compositions, their XRD profiles were refined through the Rietveld method employing the Full-prof software (version 7.95). The Rietveld results are shown in Figure 2. The XRD profiles were refined by utilizing a structural model of the BNT phase under the rhombohedral structure and space group R3c as a starting model. However, the XRD peak profiles were determined through the pseudo-Voigt function. For atomic positions, we consider that the lanthanide ions (Pr^3+^, Nd^3+^, Eu^3+^, Dy^3+^) occupy A-sites. In Figure 2, all the compositions show good correspondence between experimental intensities (red circles) and calculated intensities (black lines) with acceptable values of R-factors, which prove the R3c rhombohedral symmetry for all the prepared perovskite ceramics. According to the structural refinement results, the obtained large R-factor (Rwp) is due to the small impurity peaks that appeared at 2θ~24.66°, 27.93°, and 36.16°. For all synthesized samples, the small impurity peaks observed at 2θ~24.66° and 27.93° were attributed to the unreacted Bi_2_O_3_ and Na_2_O phases, respectively. However, the observed small XRD peak around 2θ~36.16° for both Pr and Nd-doped BNT-BT samples was identified to the Ba_1.31_Ti_8_O_16_ phase as an impurity phase. Table 1 displays the refined structural parameters of all synthesized perovskite ceramics. As clearly shown in this table, there is no significant deviation in the values of lattice parameters after doping the BNT-BT sample with different lanthanide ions. Moreover, all lanthanide ion-doping induces a small decrease in lattice parameters and volume of the unit cell. This decrement can be explicated by the difference in the ionic radii of the matrix ions ($r_{{Na}^{+}}=1.24\;Å$, $r_{{Bi}^{4+}}=1.17\;Å$, $r_{{Ba}^{2+}}=1.47\;Å$) and the doping ions ($r_{{Pr}^{3+}}=1.179\;Å$, $r_{{Nd}^{3+}}=1.163\;Å$, $r_{{Eu}^{3+}}=1.12\;Å$, $r_{{Dy}^{3+}}=1.083\;Å$). Analyzing the ionic radii reveals that the matrix ions (Na^+^, Bi^3+^, and Ba^2+^) are larger than the doping ions (Pr^3+^, Nd^3+^, Eu^3+^, Dy^3+^), which leads to a small decrement in lattice parameters. 

**Figure 3:**
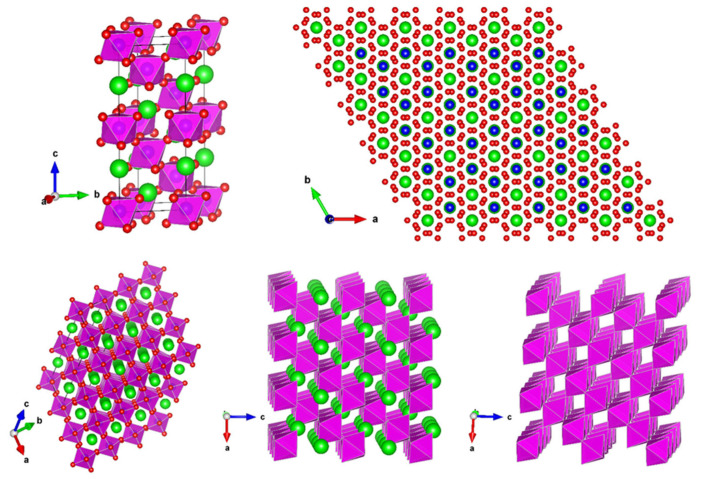
Schematic drawings of crystal structure and octahedral distortions of the synthesized Na_0.5_Bi_0.5_TiO_3_ (BNT), 0.94Na_0.5_Bi_0.5_TiO_3_-0.06BaTiO_3_ (BNT-BT), and lanthanides-doped (BNT-BT-Ln, Ln = Pr^3+^, Nd^3+^, Eu^3+^, Dy^3+^) perovskite ceramics under rhombohedral structure and space group R3c. Green spheres are used for depicting Na^+^, Ba^2+^, Bi^3+^, Pr^3+^, Nd^3+^, Eu^3+^, and Dy^3+^ cations located at A-sites, whereas the blue and red spheres represent the Ti^4+^ cations in B-sites and O^2−^ anions, respectively. TiO_6_ octahedra are depicted by pink color.

The crystal structure of the synthesized perovskites was drawn by VESTA software (Version 3) utilizing the structural parameters acquired from the Rietveld refinement. BNT, BNT-BT, and BNT-BT-Ln perovskites belong to a rhombohedral structure with the R3c space group (N° 161). Figure 3 shows their crystal structure under R3c rhombohedral symmetry. In this structure, the Na^+^, Bi^3+^, Ba^2+^, Pr^3+^, Nd^3+^, Eu^3+^, and Dy^3+^ cations in 12-coordination (green spheres) and the Ti^4+^ cations (blue spheres) are both located at the Wyckoff 6a site at 0, 0, 0.263 and 0, 0, 0.006, respectively, whereas the O^2−^ anions (red spheres) are placed at the Wyckoff 18b site (0.126, 0.336, 0.083). Figure 3 also depicts the anti-parallel distortion TiO_6_ octahedra, which may be a reason for the ferroelectric behavior. 

In addition, the stability and the crystal structure of the ABO_3_-type perovskite oxide materials can be determined by the tolerance factor (T*_f_*) using the ionic radii of cations (A^n+^, B^m+^) and oxygen anion (O^2−^), as shown in the following equation [20]:

(1) $$T_{f}=\frac{r_{A^{n+}}+r_{O^{2-}}}{\sqrt{2}(r_{B^{m+}}+r_{O^{2-}})}$$

where $r_{A^{n+}}$, $r_{B^{m+}}$, and $r_{O^{2-}}$ are the ionic radii of the cations located at A-sites. All the ionic radii were sourced from the R. D. Shannon’s ionic radii table [21]. The obtained values of tolerance factor (T*_f_*) for different compounds are listed in Table 1. Generally, for simple perovskites, when the structure is ideal cubic, the value of T*_f_* should be equal to 1. If T*_f_* > 1, the structure tends to be tetragonal, and if T*_f_* < 1, the structure would be rhombohedral [22]. In our case, all the compounds exhibit a value of T*_f_* smaller than 1, which further confirms the rhombohedral structure for all the synthesized perovskites. However, the addition of Lanthanide ions (Pr^3+^, Nd^3+^, Eu^3+^, Dy^3+^) into the BNT-BT perovskite system led to an insignificant reducing of tolerance factor value from 0.9243 to 0.9217. 

**Figure 4:**
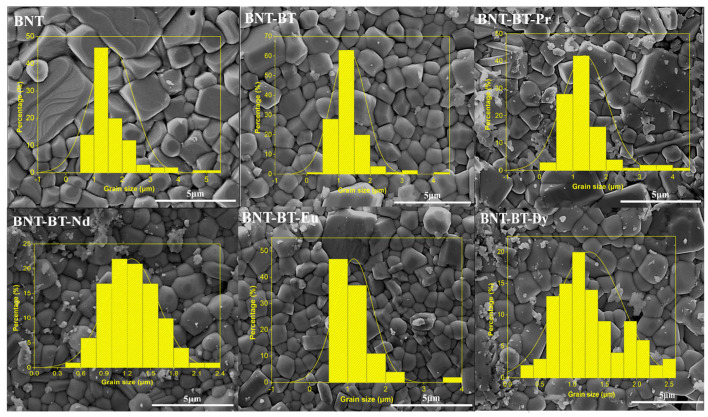
SEM micrographs and grain size of the synthesized (BNT), (BNT-BT), and lanthanides-doped (BNT-BT-Ln, Ln = Pr^3+^, Nd^3+^, Eu^3+^, Dy^3+^) perovskite ceramics.

Figure 4 shows the SEM micrographs of the prepared ceramics BNT, BNT-BT and BNT-BT-Ln. The synthesized ceramics proved to be relatively dense. The diagram in the inset of Figure 4 corresponds to the grain size distribution calculated for the BNT, BNT-BT and BNT-BT-Ln ceramics. With the addition of the lanthanides, it is observed that the average grain size considerably decreases from 1.62 to 1.15 μm as shown in Figure 4. According to previous works, the addition of lanthanide elements leads to the inhibition of grain boundary diffusion [23]. 

### 3.2. Raman Spectra

**Figure 5:**
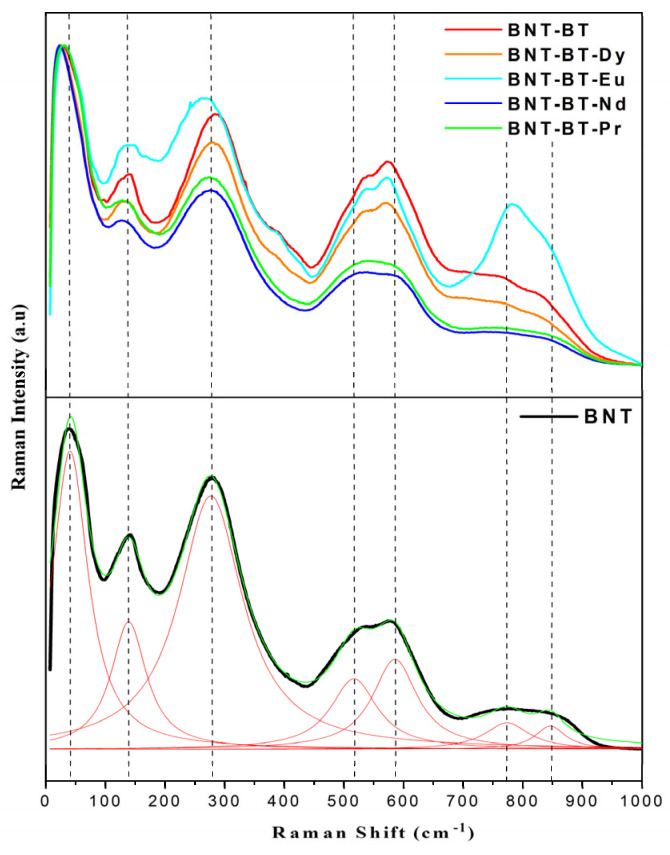
Raman spectra of synthesized ceramics at room temperature with a green laser 532 nm.

Raman spectroscopy provides more definitive insights into the microstructure and lattice vibrations of materials [24,25]. Figure 5 depicts the Raman spectrum of the BNT-BT-Ln ceramics, which was acquired on the polished surfaces of the ferroelectric ceramics to mitigate scattering effects. The spectrum of all samples, exhibit broad features due to A-site disorder, which can promote the relaxor nature in ferroelectric materials [26]. According to previous works, the Raman modes of our samples could be grouped into three domains [26]. The first domain is located at feeble wave number region (100–187 cm^−1^) which is assigned to the vibration of ions from A-site in the structure. It is caused by A-site cation (Na/Bi/Ba/Ln) variations which are very delicate in phase transitions [27,28]. The second domain, near the 187–433 cm^−1^ range, mainly arises from the internal bending and stretching vibrations of Ti–O bond. Typically, alterations in this region arise not only from the displacement of the polar Ti-cation, but additionally due to octahedral tilt and rotation distortions, which may reflect significant structural variations. This region may be useful for identifying phase transitions in both classic and complex ferroelectric materials [29]. The last domain is situated between 433 and 878 cm^−1^ and belongs to the oxygen and TiO_6_ octahedral rotations and vibrations, which are linked from superposition of the transverse optical (TO) and longitudinal optical (LO) bands characterized by A1 with the A1(LO) and E(LO) overlapping bands [30]. All Raman modes exhibit wide modes due to the increase in disorder from the incorporation of Ba (for BNT-BT) and further, the lanthanide element for the rest of the samples [31,32]. 

It seems that all Raman shifts observed modes and did not show any perceptible variation in dependence of added lanthanides, except for the BNT-BT-Eu spectrum where an enhancement of some mode intensity is observed around 800 nm. In their work about Eu^3+^-doped BNT-BT, Na Wu et al. attributed the observation of this mode to the vibrations generated by the movement of oxygen within the system caused by the creation of vacancy while substituting Bi^3+^ by Eu^3+^ [19]. However, the authors reported the disappearance of this mode with increasing Europium concentration beyond x = 0.015. In our case, if we adopt this conclusion, it is unclear why we did not observe the same phenomenon with another rare earth smaller (Dy^3+^) or larger (Pr^3+^ or Nd^3+^) than Eu^3+^ for the same concentration. We surmise then that this phenomenon could be linked to the fluorescence emission of Eu^3+^ in the NBT-BT matrix. In fact, the use of 532 nm Raman laser is able to excite the ^5^D_0_ energy level of Eu^3+^ and, consequently, luminescence occurrence [33]. However, the quenching of luminescence by the concentration effect is known, and we believe that this happened in the work reported in reference [34]. 

It is worth mentioning that the modes situated between 150 and 350 nm and 500 and 600 nm showed a slight change in their intensities by changing the nature of the lanthanide element; thus, we believe that these octahedra have undergone continuous distortion with increasing size of rare earth element.

### 3.3. Dielectric Studies

**Figure 6:**
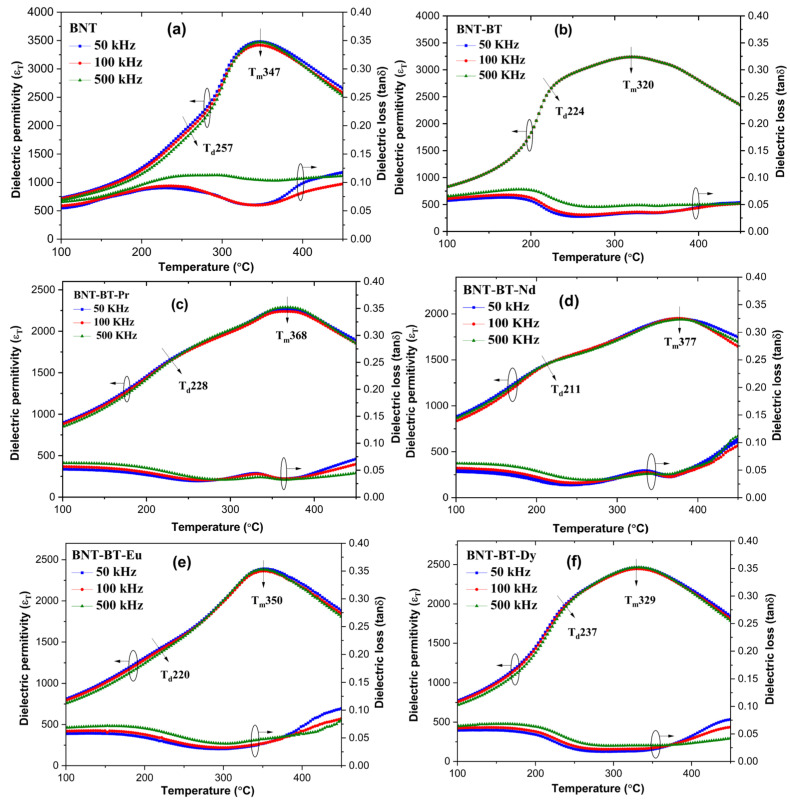
The dielectric constant and loss of (**a**) BNT, (**b**) BNT-BT, (**c**) BNT-BT-Pr, (**d**) BNT-BT-Nd, (**e**) BNT-BT-Eu, and (**f**) BNT-BT-Dy.

The impact of lanthanide ions (Nd^3+^, Pr^3+^, Eu^3+^, and Dy^3+^) on the BNT-BT matrix was investigated by the dielectric experiments at different frequencies ranging from 300 to 800 K. Figure 6 displays the changes in the relative permittivity (ε_r_) and dielectric losses (tanδ) of various ceramics with temperature. It is worth noting that the maximum dielectric permittivity value (ε_r_), for BNT and BNT-BT, which is around 3469 and 3237, respectively, is consistent with previous studies [27,34,35,36,37]. 

All the BNT-BT ceramics display two main features of dielectric permittivity [7,38]. The shoulder observed in ε_r_(T) at T_d_, was mostly attributed to depolarization temperature. In the BNT compound, Dorcet et al. [39] highlighted the existence of an additional intermediate orthorhombic structure (Pnma). The authors reported that the transition from a rhombohedral to tetragonal phase occurs via a modulated phase (R3c + Pnma), characterized by the formation of polar nanoregions (PNRs). Benyoussef et al. showed that by incorporating the Nd-element in BNT, T_d_ becomes more pronounced and accompanied with a frequency dispersion revealing the dynamics of the polar nanoregions that are generally observed in relaxor ferroelectric materials [40]. This transition was identified using the peak of dielectric loss [41]. The T_d_ has a significant impact on the usefulness of materials in real-world applications. In the case of the addition of the inclusion of Ba into such a matrix and a Ba concentration of 0.06, many studies reported the existence of morphotropic phase boundary (MPB) with the simultaneous existence of tetragonal (P4mm) and rhombohedral (R3c) crystalline phases [36,42,43,44]. 

Noting that near (T_m_), there is no significant peak shift observed at various frequencies, these peaks appear broad, indicating a diffuse phase transition. In fact, the diffuse phase transition observed in the NBT-BT system stems from the random distribution of Na^+^, Bi^3+^, and Ba^2+^ ions within the A-site [38]. 

The doped samples (BNT-BT-Ln) exhibit a shift in (T_m_) towards higher temperatures compared to the NBT-BT, which is attributable to the structural disorder [45]. Because of their small size, lanthanides really cause a significant lattice distortion when they are added to the NBT-BT matrix [46]. 

**Figure 7:**
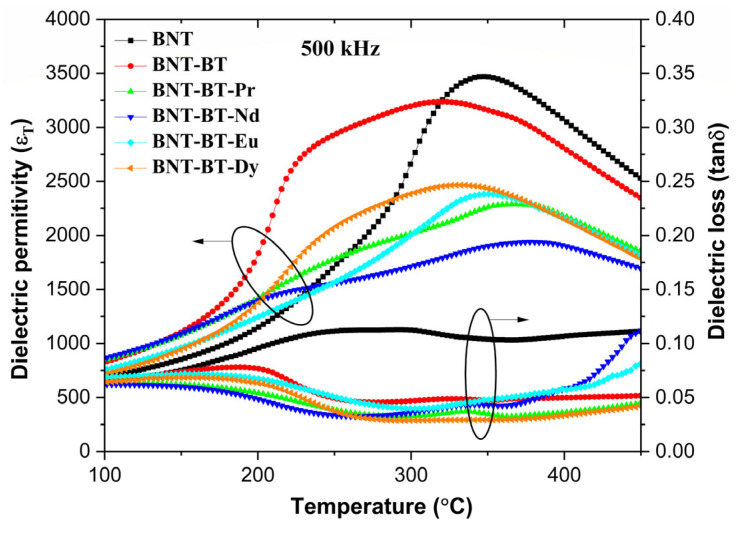
Variation of dielectric permittivity for all samples at 500 kHz as a function of temperature.

It is important to note that the dielectric permittivity of doped compositions at (T_m_) are lower than of the undoped composition. The changes in the dielectric properties are evident in Figure 7, which depicts the evolution of thermal dielectric permittivity at 500 kHz for various samples. It can be observed that the addition of rare earths into the BNT-BT causes significant alterations in these properties.

From a quantitative standpoint, adding Ln^3+^ to the NBT-BT matrix leads to a decrease in the concentration of Bi vacancies according to the following equations (using the Vink– Kroger notation): because of the volatility of Bi_2_O_3_, we expect a partial Schottky disorder according to [41,47]:

$$2\mathrm{BiBi}+3\mathrm{OO}+6h^{•}\;\to \;2V_{Bi}^{\prime \prime \prime }+{3V}_{O}^{..}+{\mathrm{Bi}}_{2}O_{3}$$

Then, the incorporation of Ln ions is on vacant Bi-sites according to:

$${\mathrm{Ln}}_{2}O_{3}+2V_{Bi}^{\prime \prime \prime }+6h^{•}\;\to \;2{Ln}_{Bi}+3/2\;O_{2}$$

Furthermore, this incorporation favorizes the development of an anti-polar phase in the system, as reported by Borkar et al., where a small amount of trivalent Al^3+^ cations is incorporated into the A site of NBT-BT system [48]. 

**Figure 8:**
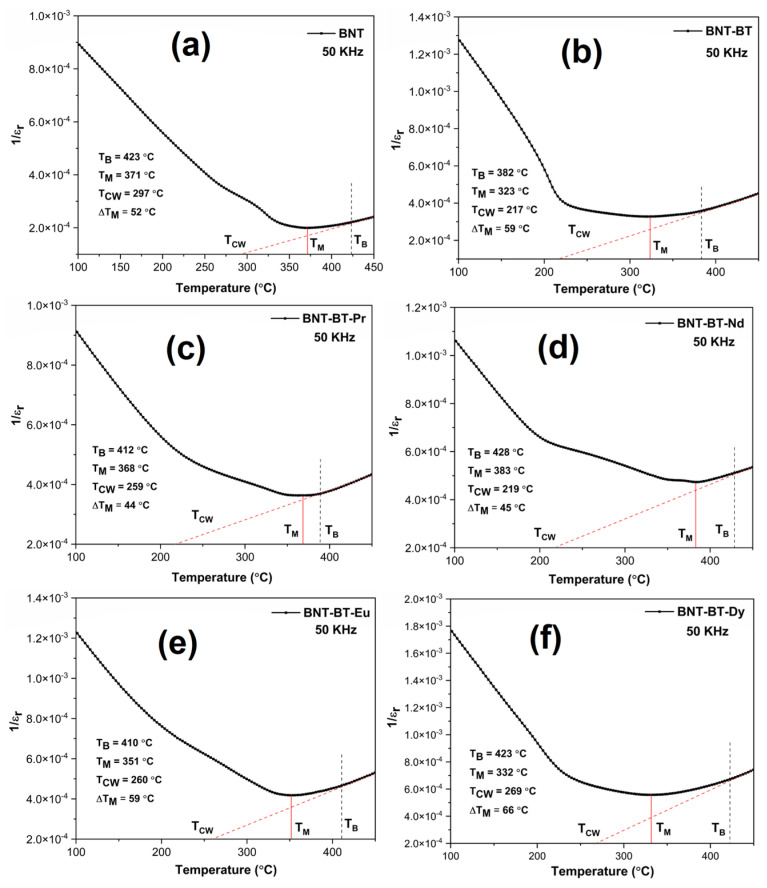
(**a**) Curve representing the diffusive behavior of (**a**) BNT, (**b**) BNT-BT, (**c**) BNT-BT-Pr, (**d**) BNT-BT-Nd, (**e**) BNT-BT-Eu, and (**f**) BNT-BT-Dy ceramics utilizing the inverse of the dielectric permittivity (1/ε_r_) versus temperature.

Figure 8 shows the curves of 1/ε_r_ as a function of the temperature at 50 kHz and correspondence with the Curie–Weiss law (CW), which generally defines the temperature dependence above the ferroelectric–paraelectric phase transition [49]:

$$\frac{1}{\varepsilon_{r}}=\frac{T-T_{CW}}{C}$$

**Figure 9:**
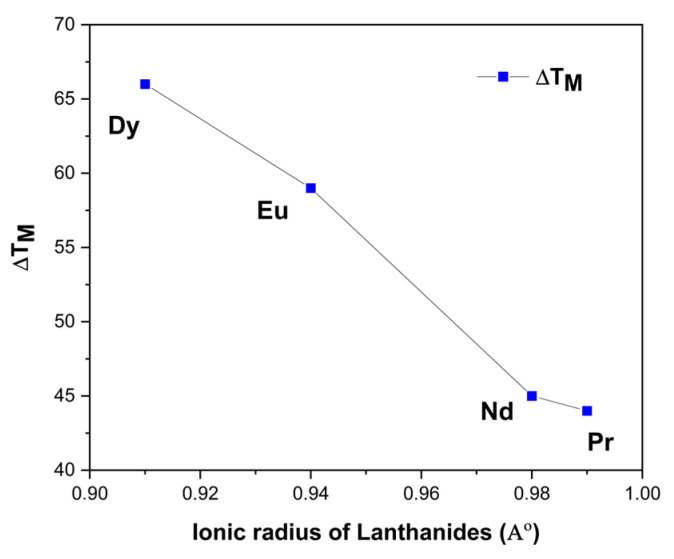
ΔT_M_ as a function of ionic radius of lanthanides.

Noting that ε_r_ is the dielectric permittivity, (T_CW_) and C are the Curie–Weiss temperature and Curie–Weiss constant, respectively. The figure shows three temperature regions, where (T_M_) is the maximum permittivity temperature, at which ε_r_(T) start to deviate from linear dependence in direction of lower temperatures (Burns temperature (T_B_)). The linear part of ε_r_(T) at high temperatures is used to determine the Curie temperature (T_cw_) [50]. The degree of deviation from the Curie–Weiss law can be quantified using ΔT_M_, which is defined as:

ΔT_M =_ T_B_ − T_M_

where (T_B_) designates the start temperature of the function whose dielectric constant conforms to the Curie–Weiss law (Burns temperature) and the temperature of the maximum dielectric constant (T_M_) [51]. As seen from Figure 8, the values of (T_CW_) for BNT-BT, BNT-BT-Pr and BNT-BT-Nd ceramics are more deviated from (T_M_) then for other compositions. Figure 9 highlights the variation of ΔT_M_ as a function of the size of the rare earth element. As depicted form the figure, the increase in ionic radius of lanthanides leads to a gradual decrease in the value of ΔT_M_; thus, a decreasing degree of diffuse phase transition in BNT-BT-Ln materials [51,52]. A summary of various obtained values is presented in Table 2.

To assess the degree of diffusivity, the modified Curie–Weiss law can be applied to describe the dielectric behavior of a relaxor ferroelectric:

$$\frac{1}{\varepsilon }-\frac{1}{\varepsilon_{m}}=\frac{{\left(T-T_{m}\right)}^{\gamma }}{C}$$

where ε is the dielectric permittivity at a particular temperature T, ε_m_ is the value at T_m_, and γ is the diffusivity. C is the modified Curie–Weiss constant.

The diffuse phase transition exhibited by the NBT-BT ceramic is indicative of relaxor behavior tendency [53], which is caused by the coexistence of cations with different valence in equivalent crystallographic sites. 

**Figure 10:**
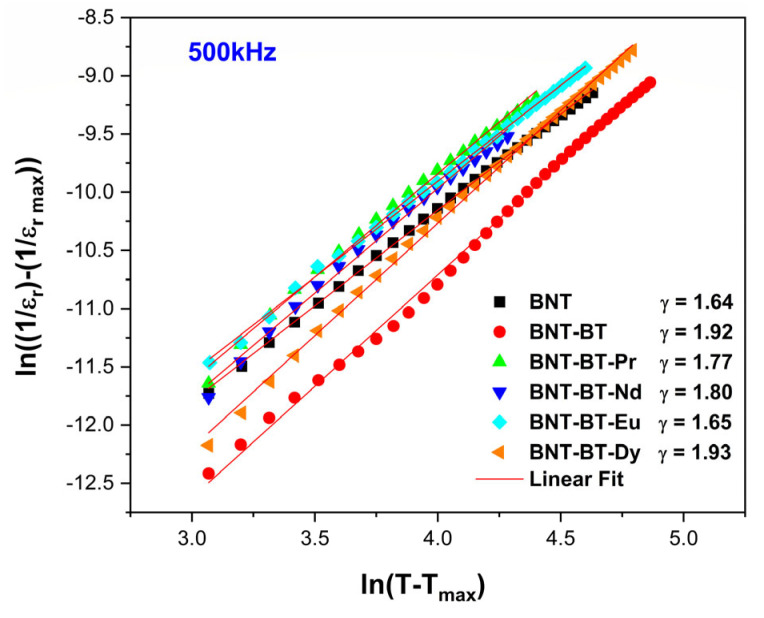
Curve depicting the variation of ln(1/ε_r_ − 1/ε_m_) as a function of ln(T − T_m_) for the synthesized ceramics.

The curve of ln(1/ε − 1/ε_m_) as a function of ln(T − T_m_) for all samples is displayed in Figure 10. The estimated values of γ were approximately 1.92 for NBT-BT, 1.77, 1.79, 1.65 and 1.93 for the samples doped with Pr_2_O_3_, Nd_2_O_3_, Eu_2_O_3_, and Dy_2_O_3_, respectively, following the implementation of a linear fit on the experimental data depicted in Figure 9. 

It is worth mentioning that a material characterized by γ = 1 is classified as a classical ferroelectric, whereas a standard relaxor ferroelectric exhibits γ = 2 [54]. The graphs illustrating ln (1/ε_r_ – 1/ε_m_) plotted against ln(T – T_m_) at 500 kHz for all compositions are shown in Figure 10, demonstrating nearly linear trends with γ values exceeding 1. This suggests that these compositions undergo a diffuse phase transition. The ceramic BNT-BT-Dy exhibited the higher value of γ (above 1.93) compared to the other doped compositions, as shown in Figure 10. 

### 3.4. Ferroelectric Performance

**Figure 11:**
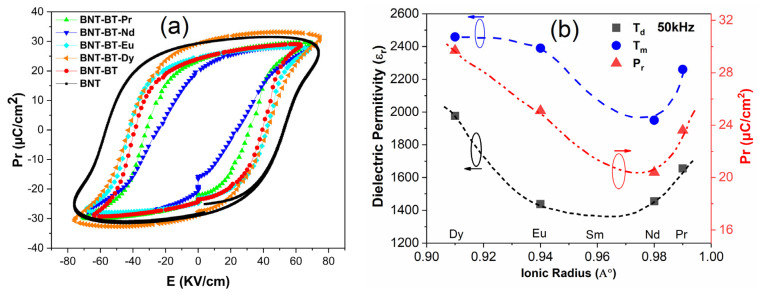
(**a**) PE hysteresis loops of all synthesized ceramics at room temperature. (**b**) Variation of some characteristic parameters versus ionic radius of the rare earth element. Lines are for visual guidance.

In Figure 11a, the hysteresis loops of the polarization electric field (PE) for all samples were analyzed at room temperature at a testing frequency of 1 Hz. It is evident that all the ceramics exhibit well-saturated ferroelectric hysteresis loops. Pure BNT-BT demonstrates a characteristic PE loop characterized by a substantial remanent polarization (Pr = 24.7 µC/cm^2^) and coercive field (Ec = 63 kV/cm), aligning well with previously reported findings [55,56].

The incorporation of Ln^3+^ elements into the BNT-BT matrix seems to have a notable impact on the characteristics of the hysteresis loop, particularly with regard to remanent polarization (Pr). Upon doping with the Dy and Eu elements, noteworthy alterations in the PE loop shape were observed, resulting in more inflated loops and a remarkable increase in remanent polarization. Specifically, the remanent polarization value increased from 24 μC/cm^2^ for BNT-BT to 29.7 μC/cm^2^ for BNT-BT-Dy. In contrast, the BNT-BT-Nd sample exhibited lower remanent polarization value Pr = 20.4 μC/cm^2^. The addition of Ln elements in the BNT-BT matrix led to increasing disorder of the A-site cations, causing a structural heterogeneity. The diminished ferroelectricity in these samples could potentially be attributed to the presence of local random fields that disrupt the long-range ferroelectric order [57]. A summary of various calculated values is presented in Table 3. Figure 11b shows the variation of T_d_, T_m_ and P_r_ versus the size of the rare earth element. It appears that these parameters decreased with decreasing lanthanide size until a minimum between Eu and Nd (probably in the Samarium element) was reached and then increased. Assuming that the Lanthanide element is located in the A-sites, the decrease in the size of such ions maintains the B–O distances, while changing the A–O distances. Such a behavior was reported to induce GdFeO_3_-type tilting BO_6_ octahedra with the change of symmetry in some perovskites [58]. On the other hand, in Ba_3_LnRu_2_O_9_ compound, Yoshihiro Doi et al. observed similar deviation in lattice parameter with the size of the lanthanide element. The authors attributed this behavior to the change in the oxidation degree state of the lanthanide (Ce, Pr and Tb) without changing the crystal structure [59]. Noting that the change in site location of the lanthanide might also lead to a change in Ln-O bond length, and thus to this behavior, more advanced investigations are necessary to understand the origin of this deviation.

**Figure 12:**
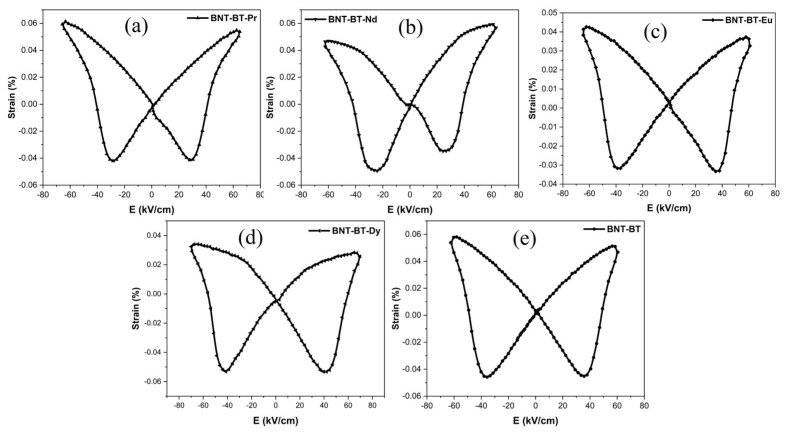
Strain curves of synthesized ceramics: (**a**) BNT-BT-Pr, (**b**) BNT-BT-Nd, (**c**) BNT-BT-Eu, (**d**) BNT-BT-Dy and (**e**) BNT-BT.

Figure 12 illustrates the S-E (strain-electric field) loops induced by bipolar electric fields in Ln-BNT-BT ceramics at room temperature. These S-E curves exhibit the classic butterfly shape typical of ferroelectric materials [60,61].

However, when lanthanides are introduced, this butterfly shape gradually undergoes alterations. Notably, the “positive strain” diminishes progressively, particularly for BNT-BT-Nd, BNT-BT-Eu, and BNT-BT-Dy compositions.

At room temperature, the highest positive strain (Smax) of 0.062% is observed in the case of BNT-BT-Pr, while the most substantial negative bipolar strain of 0.053% is recorded for BNT-BT-Dy. The latter can be related to the more stable domain structure in BNT-BT-Dy in respect to the applied field, considering also the PE loop segment, where direction of spontaneous polarization and applied field are opposites. Just opposite of the direction of total polarization and applied field is the reason of negative strain. Considering that absolute value of strain is proportional to the product of both quantities, lower concentration of domains, which are switched in the field direction in this loop segment, will lead to higher polarization and higher values of negative strain.

Such an assumption is consistent with the observed lower values of dielectric permittivity at room temperature for Dy-doped composition.

### 3.5. Photoluminescence (PL) Investigations

**Figure 13:**
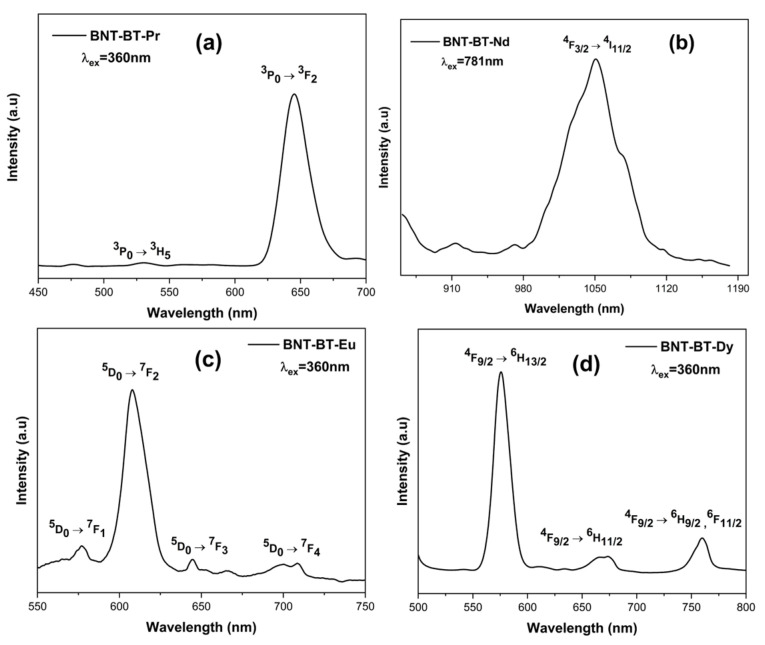
Emission spectra of (**a**) BNT-BT-Pr, (**b**) BNT-BT-Nd, (**c**) BNT-BT-Eu and (**d**) BNT-BT-Dy samples excited with appropriate wavelengths.

The photoluminescence investigation on the BNT-BT-Pr, BNT-BT-Nd, BNT-BT-Eu, and BNT-BT-Dy compounds under various excitation wavelengths is shown in Figure 13.

Using a 360 nm laser, the Pr^3+^-doped BNT-BT composition was excited at room temperature. The emission spectra of Pr^3+^-doped BNT-BT exhibit a central red emission at 644 nm, which corresponds to the transitions from ^3^P_0_ to ^3^F_2_, similar to other Pr^3+^-doped perovskite materials [62,63]. The second emission, occurring at 530 nm, is associated with the transition ^3^P_0_ → ^3^H_5_, which results in a modest emission of green light. Our results are in agreement with earlier studies [64]. 

A 781 nm wavelength was used to excite the Nd^3+^-doped BNT-BT ceramic, which then radiated in the near infrared. A prominent band at 1050 nm, which corresponds to the transition of ^4^F_3/2_ → ^4^I_11/2_, is detected in the BNT-BT-Nd spectra (Figure 13). This emission band was also noted in a previous publication by Robin et al. [65]. 

At 360 nm excitation, a prominent red emission peak at 607 nm, which is associated with the ^5^D_0_ → ^7^F_2_ transition, dominates the PL spectra of the BNT-BT-Eu^3+^. The transitions ^5^D_0_ → ^7^F_1_, ^5^D_0_ → ^7^F_3_, and ^5^D_0_ → ^7^F_4_ are represented by the comparatively feeble emissions with peaks at 576, 644, and 704 nm, respectively [66].

Three distinctive emission peaks at 575 nm, 669 nm, and 760 nm are found in the emission spectra of the compound BNT-BT-Dy (Figure 13) and are attributed to the Dy^3+^ transitions ^4^F_9/2_ → ^6^H_13/2_, ^4^F_9/2_ → ^6^H_11/2_ and ^4^F_9/2_ → ^6^H_9/2_, and ^6^F_11/2_, respectively [67]. Our results are well in line with those of Kuzman et al. [68] and Ma et al. [69].

**Figure 14:**
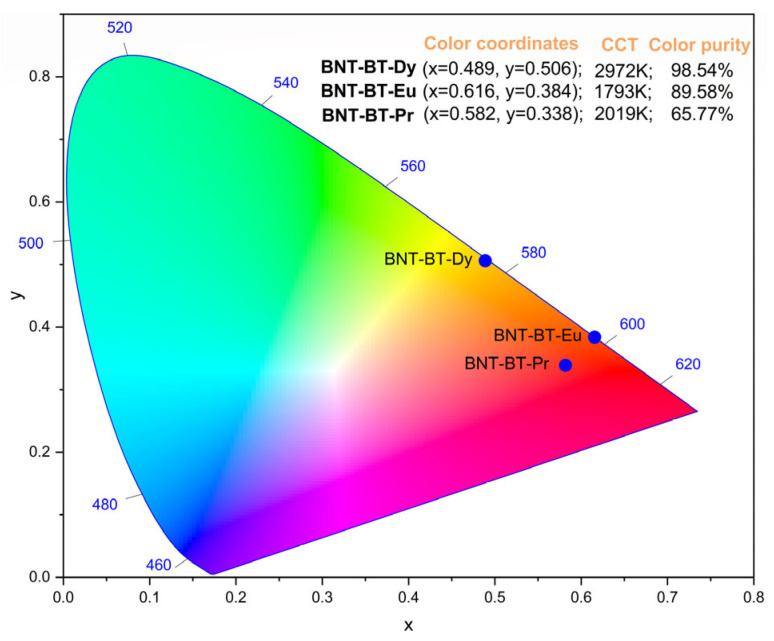
The CIE chromaticity diagram for BNT-BT-Pr, BNT-BT-Eu and BNT-BT-Dy.

The chromaticity diagrams based on the “International Commission of Lighting” (CIE) 1931 standards [70], correlated color temperature (CCT) values, and color purity for BNT-BT-Pr, BT-BT-Eu, and BNT-BT-Dy are illustrated in Figure 14. These parameters are crucial for assessing the material’s performance in terms of color luminescent emission, particularly in practical applications like light-emitting diodes (LEDs). The CCT values are computed with the CIE 1931 web-based app [71,72] using the McCamy empirical relation [73] and are presented as follows:

$$CCT\left(x,y\right)={-449n}^{3}+{3525n}^{2}-6823.3n+5520.33$$

In the given expression, where $n=\frac{\left(x-x_{e}\right)}{\left(y-y_{e}\right)}$, the coordinates (x, y) represent the color coordinates of a sample, while (*x_e_* = 0.3320, *y_e_* = 0.1858) denote the epicenters of the convergence.

The color purity of the emitted color in the BNT-BT-Pr, BNT-BT-Eu, and BNT-BT-Dy systems is determined through the application of the formula provided in [74]:

$$\mathrm{Color}\;\mathrm{purity}=\frac{\sqrt{{\left(x-x_{s}\right)}^{2}+{\left(y-y_{s}\right)}^{2}}}{\sqrt{{\left(x_{d}-x_{s}\right)}^{2}+{\left(y_{d}-y_{s}\right)}^{2}}}\times 100$$

where:

x and y represent the CIE coordinates of the entire spectrum.
x_s_ and y_s_ denote the the CIE coordinates of the standard illuminants of white light.
x_d_ and y_d_ stand for the CIE coordinates of the dominant wavelength.

Observing Figure 14, it is evident that the CIE color coordinates for the BNT-BT-Pr, BT-BT-Eu, and BNT-BT-Dy systems (x = 0.582, 0.616 and 0.489) are situated comfortably within the red region. The corresponding CCT values are reported as 2019 K, 1793 K, and 2972 K, respectively. Notably, all ceramics exhibit high color purity, making them promising candidates for solid-state lighting applications.

## 4. Conclusions

0.94Na_0.5_Bi_0.5_TiO_3_-0.06BaTiO_3_ doped with Ln^3+^ (Pr^3+^, Nd^3+^, Eu^3+^, Dy^3+^) were prepared using the solid-state technique. Analysis by XRD confirmed that all samples showed pure phase with rhombohedral R3c symmetry, albeit a small impurity could be detected with very low intensity. SEM images demonstrated that the synthesized ceramics were homogeneous and uniform. The temperature dependence of dielectric permittivity indicated similar transition temperatures but with some variations in the dielectric constants after incorporating lanthanide elements into the BNT-BT matrix. Only Dy-based composition undergoes a diffuse phase transition with high coefficient of diffusivity (γ) compared to the mother BNT-BT phase. Furthermore, all the examined samples exhibited saturated hysteresis loops that changed with the nature of the lanthanide element. It is highlighted in this work that Dy incorporation led to a comparable dielectric behavior as the mother BNT-BT matrix but with improved ferroelectric properties. 

Piezoelectric properties have been performed by the mean of the strain-electric field; the results demonstrated well-expressed loops of butterfly shape, characteristic of ferroelectric states. The ratio of positive and negative strains varies in dependence of the doping element. 

Furthermore, the inclusion of Ln^3+^ within the BNT-BT ceramic resulted in distinct light emission in the visible and near-infrared regions upon exposure to different excitation wavelengths. The concurrent presence of ferroelectric and optical properties in this system presents considerable potential for optoelectronic applications.

## Figures and Tables

**Table 1:** Unit cell parameters (a, c, and V) and the value of tolerance factor (T*_f_*), reliability factor R_wp_, and goodness (χ^2^) of the synthesized Na_0.5_Bi_0.5_TiO_3_ (BNT), 0.94Na_0.5_Bi_0.5_TiO_3_-0.06BaTiO_3_ (BNT-BT), and lanthanides-doped (BNT-BT-Ln, Ln = Pr^3+^, Nd^3+^, Eu^3+^, Dy^3+^) perovskite ceramics.

Sample	BNT-BT-Ln (Ln = Pr^3+^, Nd^3+^, Eu^3+^, Dy^3+^)							
	Structure	Space Group	a (Å)	c (Å)	V (Å^3^)	*T_f_*	*R_wp_*	*χ* ^2^
BNT	Rhombohedral structure	R3c	5.4991 (51)	13.4367 (22)	351.89 (56)	0.9187	10.43	1.38
BNT-BT			5.4884 (25)	13.5503 (67)	353.49 (29)	0.9243	11.47	1.32
BNT-BT-Pr			5.5005 (08)	13.4353 (39)	352.03 (13)	0.9220	9.87	1.29
BNT-BT-Nd			5.4993 (09)	13.4420 (46)	352.05 (14)	0.9220	11.96	1.31
BNT-BT-Eu			5.4920 (06)	13.4426 (12)	351.14 (06)	0.9218	10.28	1.25
BNT-BT-Dy			5.5028 (06)	13.4572 (11)	352.90 (16)	0.9217	12.17	1.43

**Table 2:** Obtained values T_B_, T_M_, T_CW_ and ΔT_M_ of all samples.

Samples	T_B_ (°C)	T_M_ (°C)	T_CW_ (°C)	ΔT_M_ (°C)
BNT	423	371	297	52
BNT-BT	382	323	217	59
BNT-BT-Dy	423	332	269	66
BNT-BT-Eu	410	351	260	59
BNT-BT-Nd	428	383	219	45
BNT-BT-Pr	412	368	259	44

**Table 3:** Evolution of ferroelectric coefficients of BNT-BT, BNT-BT-Ln and BNT.

Samples	Pr (μC/cm^2^)	E (kV/cm)
BNT	29.5	73.6
BNT-BT	24.7	63
BNT-BT-Dy	29.7	74.8
BNT-BT-Eu	25.1	67.5
BNT-BT-Nd	20.4	67.1
BNT-BT-Pr	23.6	69.8

## Data Availability

Data are contained within the article.
